# Successful Transplantation of Pediatric Kidneys Despite Vascular Injuries

**DOI:** 10.7759/cureus.3073

**Published:** 2018-07-30

**Authors:** George Rofaiel, Sridhar R Allam, Mohammed Ali, Eryberto Martinez, Chase T Brower, Sameh A Fayek

**Affiliations:** 1 Surgery, University of Utah School of Medicine/Huntsman Cancer Institute, Salt Lake City, USA; 2 Transplantation Surgery, Medical City Fort Worth, Fort Worth, USA; 3 Surgery, Medical City Fort Worth, Fort Worth, USA; 4 Transplant Surgery, University of Utah, Salt Lake City, USA

**Keywords:** kidney transplant, vascular injuries, pediatric donors

## Abstract

The gap between the kidney transplant recipient list and the number of organs available for transplantation continues to grow. Pediatric donors help fill a small and valuable portion of that gap. Normally these organs are transplanted en-bloc by closing the proximal vascular caps and using the distal aorta and distal inferior vena cava (IVC) for inflow. They are however commonly injured during the donor operation making the standard operation for pediatric en-bloc transplantation not possible. This case report presents two cases in which injured small pediatric kidneys were transplanted successfully in adult patients. We are presenting two examples of common vascular injuries to small pediatric kidneys, one venous and one arterial. In both scenarios, the kidneys were transplanted using a modification to the standard technique. The two kidneys were separated and the technique of implantation was modified to allow safe transplantation. This way we were able to transplant both kidneys successfully and using a reproducible methodology. Both recipients were young adults. There were no surgical complications.

## Introduction

There is a large discrepancy between the number of organ donors and the number of patients on the kidney transplant list. As of June 18, 2018, over 95,000 patients are on the waiting list for a kidney transplant in the United States [1]. The growth in kidney transplantation has been sluggish, with only roughly 20,000 cases performed in 2017 [2]. The number of living-donor procedures performed has also remained stagnant for several years [3]. Patients who are unable to receive transplants face high mortality and morbidity related to end-stage renal disease (ESRD) needing dialysis. Pediatric donors provide a resource to increase the number of kidneys available for transplant. As a group, they have also proven to provide equivalent [4, 5] or superior [6, 7] long-term outcomes to standard adult kidneys. The most concerning complication is early loss due to vascular thrombosis—which appears to be more frequent when kidneys from this population are used. In addition, these kidneys are commonly injured during procurement [8]. In general, repair is not attempted due to an elevated risk of vascular complications.

We present two cases of successful transplantation of small pediatric kidneys with major vascular injuries. Such injuries occur in 18–36% of all donors. Performing the multi-organ donor operation in donors of such small body size is very challenging—explaining the high rates of injury [9, 10]. We modified the standard technique of pediatric en-bloc transplantation to mitigate the injuries in two instances. This was achieved by stacking both pediatric kidneys on the same side. We achieved estimated glomerular filtration rates (eGFR) that were as expected for the size of the kidneys. In both cases, the improvements in eGFR were consistent with the normal pattern observed following the standard technique.

## Case presentation

Case 1

**Figure 1:**
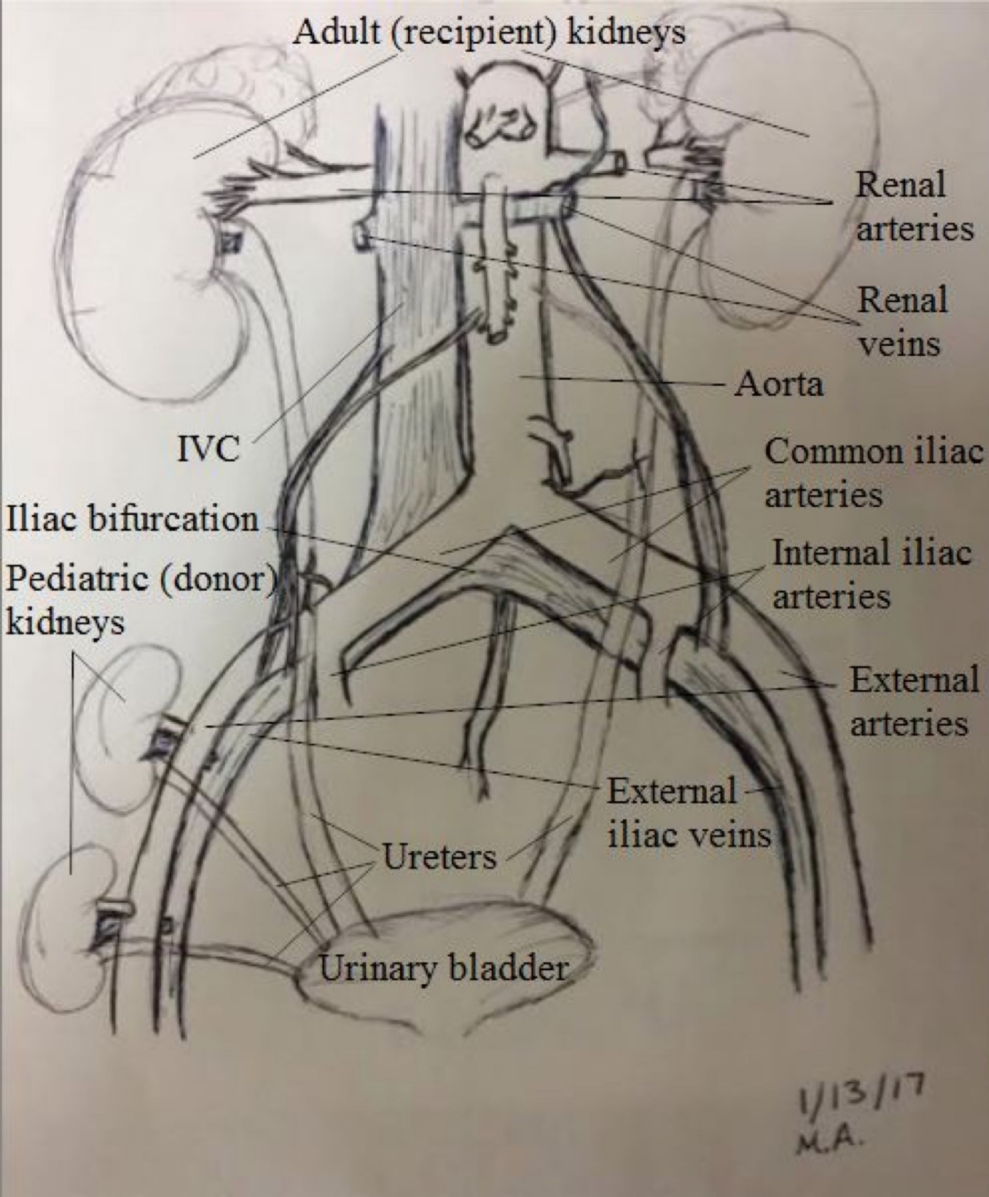
Schematic presentation of the operation. IVC: Inferior vena cava

**Figure 2:**
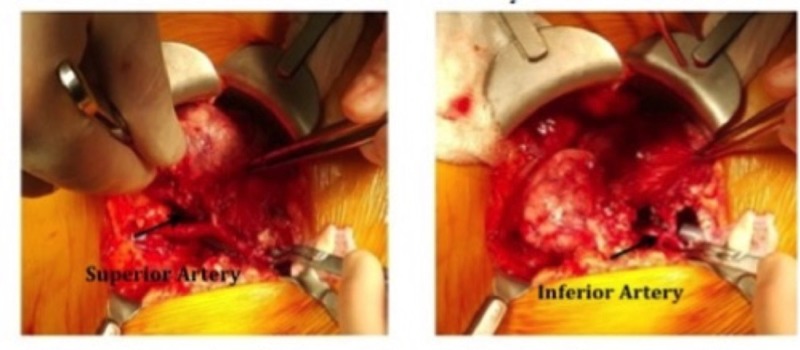
Intraoperative picture of first case.

**Figure 3:**
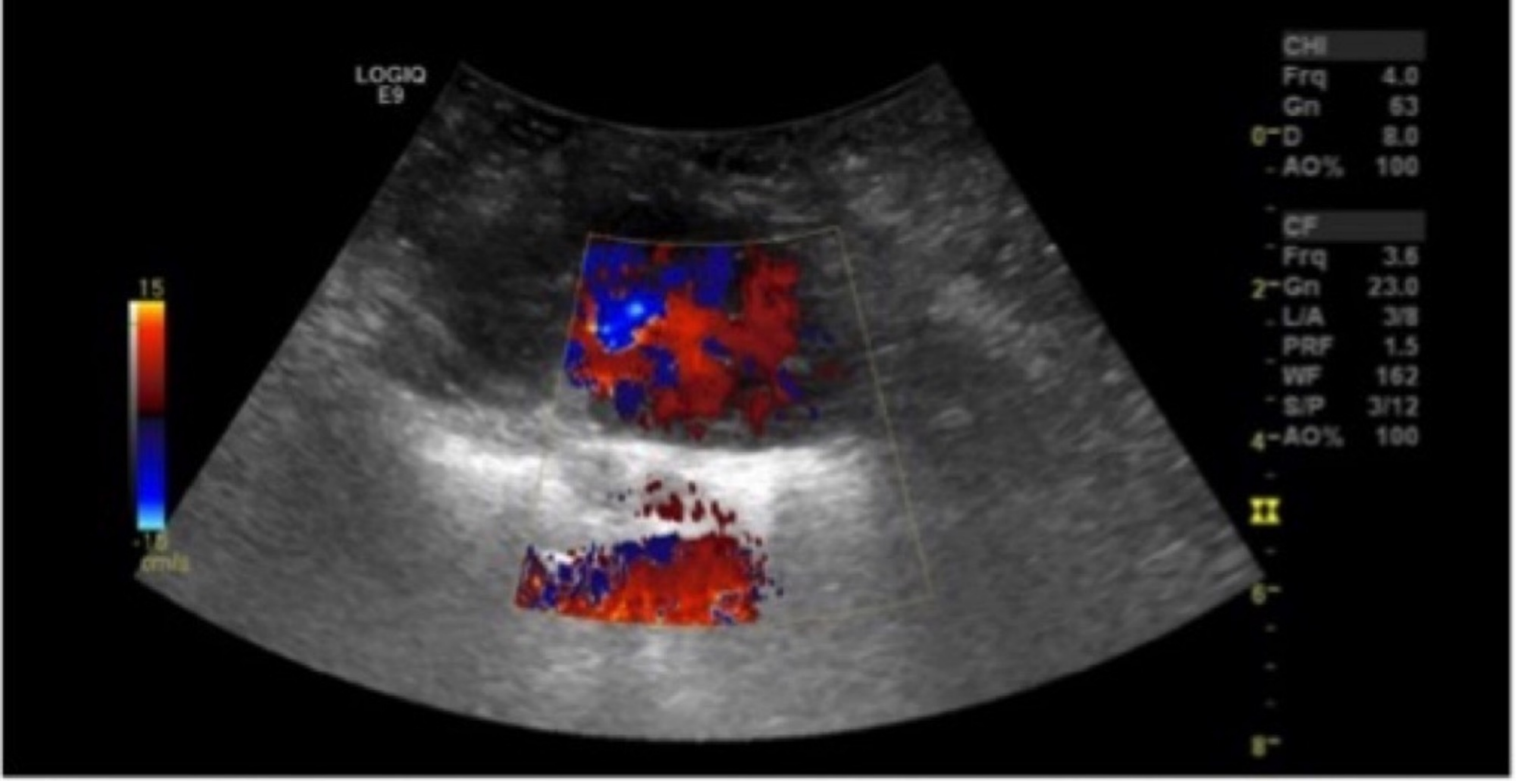
Immediate postoperative ultrasound (US).

The patient in this study is a 23-year-old female with ESRD secondary to hypoplastic kidneys. She received an offer from a brain dead, 9-month-old female donor with a terminal creatinine of 0.2 mg% and donor weight of 10 kilograms. The recipient-to-donor weight ratio was roughly six-to-one. After performing the standard multi-organ procurement, it was noted that the donor left renal vein was essentially dismembered from the inferior vena cava (IVC). This prohibited performing the standard en-bloc technique to transplant the kidneys. Normally, the suprarenal IVC is sutured closed, and the infrarenal IVC is spatulated at the iliac bifurcation for anastomosis to the recipient iliac vein. The pediatric kidneys were divided on the back table similar to an adult kidney procedure. The left renal vein was separated completely. The aorta was split down the middle and Carrel patches were made around the renal arteries. The right renal vein was elongated using the donor IVC in the standard fashion, and a venotomy was made on the side of the IVC for the anastomosis. The transplant then proceeded normally by exposing the right iliac vessels. Adequate exposure was achieved. The internal iliac veins were ligated and divided to elevate the external iliac vein and allow for a tension-free anastomosis. The external iliac artery was dissected to its full length, from the takeoff of the internal iliac artery to the inguinal ligament. The two kidneys were transplanted by stacking them on the same side. The left kidney was placed higher to allow the longer vein to cross over the iliac artery. The arterial anastomosis was performed using Carrel patch to the external iliac artery. The iliac veins were reconstructed in the usual fashion to the external iliac veins. Schematic diagram of the techniques is demonstrated in Figure 1. We used 7.0 non-absorbable monofilament stitches in a running fashion for all anastomoses. Both kidneys were reperfused simultaneously (Figure 2). The ureters were then reconstructed using two separate Lich-Gregoir anastomoses to the bladder. The total cold ischemia time was 22 hours and 47 minutes. The warm ischemia time was 55 minutes. Immediate postoperative ultrasound demonstrated excellent perfusion in both kidneys (Figure 3). The kidneys had immediate function with excellent postoperative urine output and no dialysis needs.

**Figure 4:**
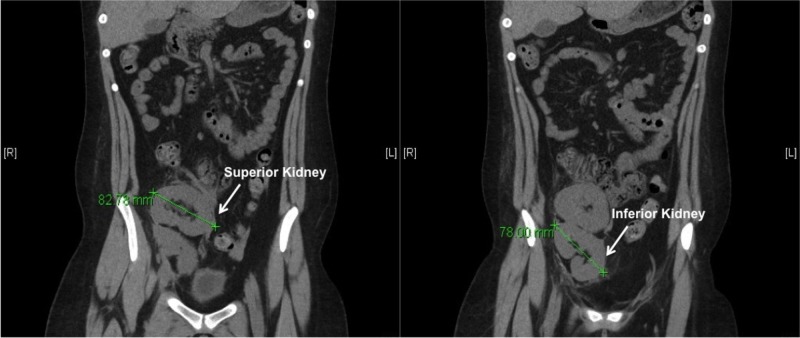
Computed tomography (CT) scan a year later showing significant growth from a baseline length of about 4.5 cm to about 8 cm.

The patient’s initial post-transplant eGFR was 9 ml/min, and had a creatinine of 6.4 mg/dL and eGFR of 10 ml/min on post-operative day one. On post-operative day six, the patient was discharged with a baseline creatinine of 1.3 mg/dL. The eGFR continued to improve to 82 ml/min 30 days post-transplant, reaching a maximum eGFR of 94 ml/min at 30 weeks post-transplant. About a year later the patient had a computed tomography (CT) scan for an unrelated reason and it showed significant growth in the size of the kidneys (Figure 4). The patient continues to maintain the same eGFR over two years later despite an episode of BANF IIA acute cellular rejection (ACR) and antibody-mediated rejection (AMR) a few months after the transplant. This episode of ACR/AMR was treated using rabbit antithymocyte globulin followed by total plasma exchange and intravenous immunoglobulin (IVIG) and rituximab. She required several biopsies throughout the course of this episode, which were technically easy to perform using standard ultrasound-guided technique.

Case 2

**Figure 5:**
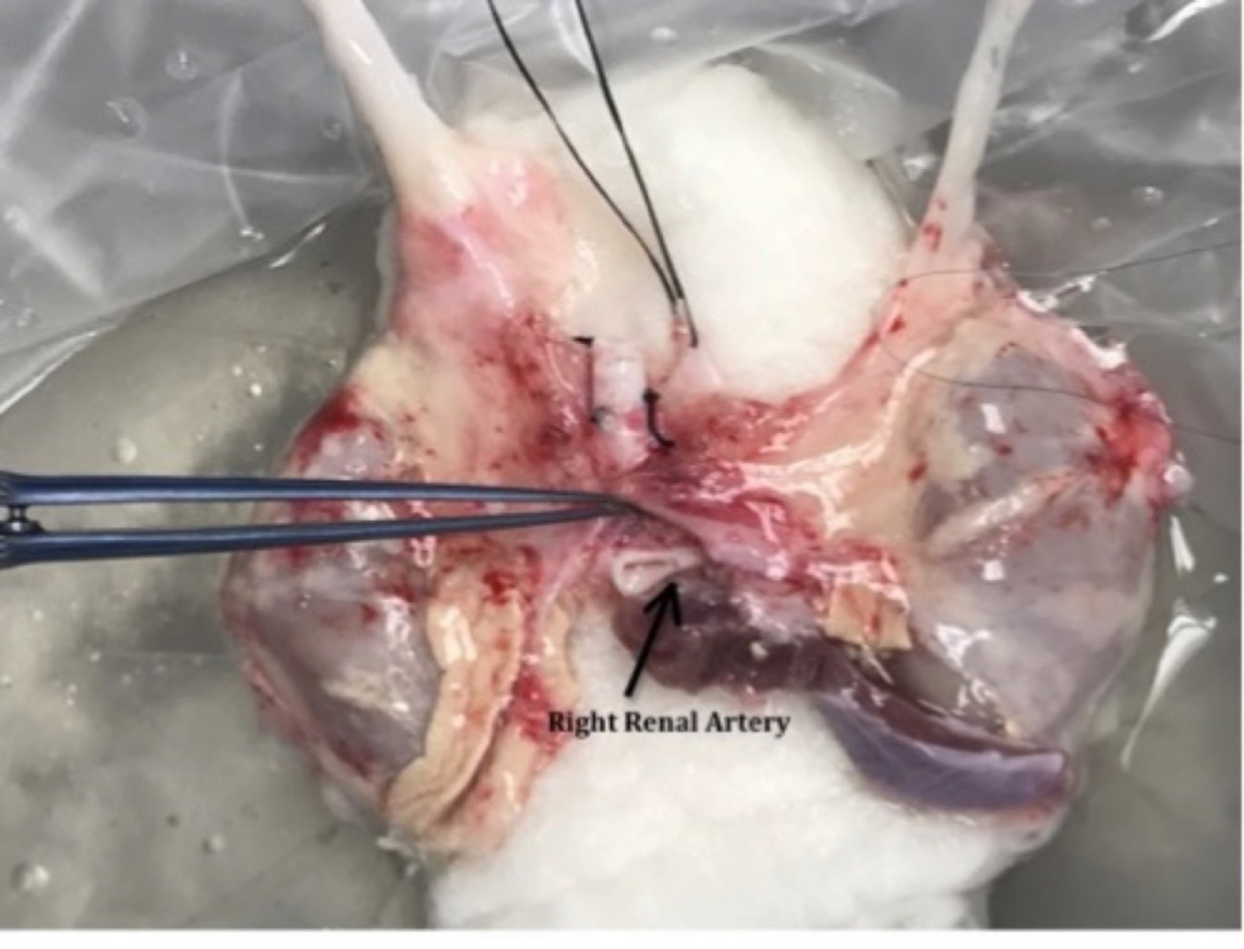
Back table picture showing arterial injury.

The second patient was a 36-year-old male who had ESRD secondary to adult polycystic kidney disease. He received an offer from a 6-month-old brain dead male donor who weighed seven kilograms. The donor had a terminal creatinine of 0.4 mg%. The recipient-to-donor weight ratio was nearly ten-to-one. After the multi-organ procurement operation was performed, it was noted that the suprarenal aorta was cut too close to the right renal artery (Figure 5). The upper half of the circumference of the artery had no aortic cuff. A concept similar to the one used in the first case was utilized. However, during this procedure, the right renal artery was reconstructed using interrupted 8.0 non-absorbable monofilament stitches rather than using a Carrel patch. The remainder of the operation was similar in technique to the prior scenario. The patient had an excellent post-transplant outcome and was discharged on the fourth postoperative day—with an eGFR of 42 mL/min, and eGFR of 86 mL/min nine months post-transplant. There are no vascular complications to date. The patient maintains his eGFR today—nearly two years later.

## Discussion

Kidneys from small pediatric donors are an option to increase the number of organs available for transplantation. They have shown good outcomes, with function and rejection rates similar to adult kidneys. Injuries to the pediatric en-bloc grafts are very common. Alternative surgical techniques to mitigate such injuries, with at least equivalent success, were attempted in this report. To our knowledge, this is the first time this technical modification has been described to mitigate vascular injuries that occur secondary to the donor operation in small pediatric donors. Shapiro et al. reported a technique of reconstructions using an adult iliac vein for vein injuries [11]. In that operation, he used a cadaveric graft to reproduce the anatomy of a standard en-bloc technique. Although his technique seems feasible, it adds several suture lines and requires a very extensive reconstruction that is inherently liable to surgical complications. Several strategies have been described to repair arterial injuries in kidneys harvested from adult donors [12], but not from pediatric donors. The technique we described in this paper is simple and reproducible. Grafts or vessels were also not required for our operation.

Another observation noted in these two cases is that the eGFR equalizes rather rapidly, regardless of the recipient-to-donor weight ratio. This observation is being described in a much larger study that is currently under review for publication, but has also been reported by others [13].

## Conclusions

This operation uses a simple modification to the standard transplant technique by stacking the kidneys on one side to achieve a safe transplant with no major vascular reconstructions. These cases provide an opportunity to explore transplantation of kidneys from small pediatric donors with vascular injuries. The outcomes were excellent, with no early or medium-term complications related to the technique. Further studies will be required to validate the reproducibility of this method.
